# Editorial: Translational Epilepsy: An Experimental to Clinical Update

**DOI:** 10.3389/fneur.2022.939559

**Published:** 2022-05-25

**Authors:** Mohd Farooq Shaikh, Ayanabha Chakraborti, Terence John O'Brien, Azman Ali Raymond

**Affiliations:** ^1^Neuropharmacology Research Laboratory, Jeffrey Cheah School of Medicine and Health Sciences, Monash University Malaysia, Bandar Sunway, Malaysia; ^2^Department of Neuroscience, Central Clinical School, Monash University, Melbourne, VIC, Australia; ^3^Department of Surgery, The University of Alabama at Birmingham, Birmingham, AL, United States; ^4^Department of Medicine, Faculty of Medicine, Jalan Hospital, MARA University of Teknology, Shah Alam, Malaysia

**Keywords:** epilepsy, translational, experimental, clinical, neurology

Epilepsy is a chronic neurological disease and people with epilepsy have an increased risk for cognitive, behavioral, and psychosocial disorders that can adversely impact the quality of life. To date, there are over 25 anti-epileptic drugs in the clinical use, but despite this still at least 30% of patients fail to achieve seizure control. Both experimental and clinical studies are important to not only understand the drug targets and disease mechanisms but also to discover more potent, safe and efficacious drugs.

The aim of this Research Topic is to highlight advances in the field of translational epilepsy research which involves basic, clinical and translational research in epilepsy and other seizure disorders. Translational research is an important aspect of epilepsy research and aims to play a key role in improving the quality of life for people with epilepsy. It is also expected to help in understanding the complexity of the disease condition and of its comorbidities.

This Research Topic compiles six articles, including one review, two mini-review, and three original research articles from prominent scientists in the field. This compilation of papers comprehensively covers translational research in epilepsy, from insights into molecular mechanisms to clinical studies. The articles also highlight the importance and necessity of a standardized diagnostic tool to explicate any mechanistic insights that could help fill in the gaps in the underlying mechanism of the disease as well as to improve the quality of the patients' life. Perspectives on translational research in this area through clinical samples and animal models are covered as well as novel findings that are expected to assist in grasping the complexity of the disease conditions and its comorbidities are also highlighted in many of the papers. The content of each article is summarized below.

A systematic review by Ngadimon et al., oversees the relation between post-traumatic epilepsy (PTE) and cognitive impairment post-traumatic brain injury (TBI) based on clinical studies. This review specifically highlights a crucial need for a standard cognitive assessment tool to further solidify the link between patients with PTE and poor cognitive performance.

A mini-review by Beltrán-Corbellini et al., covers a comprehensive issue on epilepsy genetics and precision medicine in adult patients specifically on the developmental and epileptic encephalopathies (DEEs). This review highlights the importance of a proper diagnostic approach for early diagnosis and assessment of phenotypes in adult patients for the development of targeted therapies.

In another mini-review by Chen et al. analyzed the role of aberrant hippocampal neurogenesis in epilepsy. Their findings highlight the potential role of neural stem cells (NSCs) as a targeted treatment for epilepsy. Furthermore, advances and the potential of the epilepsy-in-a-dish model were also covered to further bring about the molecular mechanism of epilepsy and develop precision therapies in epileptic patients.

Liu and Zhang, in their original research article, developed a mouse model of extended hippocampal kindling to explore the existence of seizure clusters in spontaneous recurrent seizures (SRS). The authors speculate that a systemic homeostatic mechanism further than the proposed forebrain network activity could play a vital role in regulating the occurrence and termination of seizure clusters in hippocampal kindled mice. Although more optimized experimentation and continuous monitoring are highly needed, this finding could deepen the perception of seizure occurrence patterns to improve the management of epileptic seizures and the effects of antiepileptic manipulation.

A coding for the protein Caspr2 (CNTNAP2) plays an important role in the balance between excitatory and inhibitory postsynaptic currents (E/I balance). An imbalance of E/I currents could lead to a lot of circuit dysfunction and diseases namely, epilepsy, depression, anxiety, and autism spectrum disorder (ASD). In this original research article, Lu et al. discover a novel pathogenic missense mutation in the CNTNAP2 gene in an infant with SRS and intellectual disability. Named CNTNAP2 R777G, this mutated gene shows decreased or weakened spontaneous excitatory postsynaptic currents and inhibitory post-synaptic currents as well as, impaired action potential. Although the findings are limited to small gene samples from family members, this exciting finding could open a new door to further understand the molecular mechanism of SRS.

The studies by Khatoon et al. aimed to explore the neuroprotective effect of fisetin using pentylenetetrazol-induced kindling in mice. Fisetin, a flavonoid that has been proven to possess neuroprotective properties was further analyzed to look at its role in targeting inflammatory and apoptotic mediators related to the pathogenesis of seizures. They reported that fisetin has potential anti-seizure effect against both generalized and absence seizures and probable anti-inflammatory effect that contribute to its neuroprotective effect against seizure activity through suppression of the IL-1R/TLR axis. Furthermore, downregulation of cytochrome C and caspase-3 expression was observed in kindled mice administered with fisetin. These findings can be further studied for their anti-inflammatory mechanisms to lay the foundations for newer research areas in the treatment of neurological diseases.

The editorial team is appreciative of all the authors, scientists and investigators for their contributions to the special Research Topic. We are confident that these diverse, interesting and important papers will guide researchers in their future research and will kindle advanced discussions in translational epilepsy research.

## Author Contributions

MS took the initiative for the editorial write-up. AC, TO'B, and AR also contributed to writing, revising, and proofreading. All the authors approved the editorial.

## Conflict of Interest

The authors declare that the research was conducted in the absence of any commercial or financial relationships that could be construed as a potential conflict of interest.

## Publisher's Note

All claims expressed in this article are solely those of the authors and do not necessarily represent those of their affiliated organizations, or those of the publisher, the editors and the reviewers. Any product that may be evaluated in this article, or claim that may be made by its manufacturer, is not guaranteed or endorsed by the publisher.

